# Evaluating total tumor load (TTL) to predict metastasis in four or more axillary lymph nodes in HR +/HER2- breast cancer patients: the TULIP4N + study

**DOI:** 10.1007/s12094-026-04426-1

**Published:** 2026-06-06

**Authors:** Esther Zamora, Antonio Piñero-Madrona, Isabella Sperduti, Anna Sapino, José Luis Fougo, Vicente Peg

**Affiliations:** 1 Medical Oncology Department, Vall d’Hebron University Hospital—Vall d’Hebron Institute of Oncology (VHIO), Barcelona, Spain; 2 Breast Surgery Unit, Virgen de la Arrixaca University Hospital, Murcia, Spain; 3 Department of Surgery, Pediatrics, Obstetrics and Gynecology, University of Murcia, Murcia, Spain; 4 Biostatistics Unit, IRCCS Regina Elena National Cancer Institute, Rome, Italy; 5 Candiolo Cancer Institute, FPO-IRCCS, Candiolo, Italy; 6 Department of Surgery and Physiology, Faculty of Medicine, University of Porto, Porto, Portugal; 7 Breast Center, Centro Hospitalar Universitário São João, Porto, Portugal; 8 Pathology Department, Vall d’Hebron University Hospital, Barcelona, Spain; 9 Morphological Sciences Department, Universidad Autónoma de Barcelona, Bellaterra, Barcelona, Spain

**Keywords:** Total tumor load, OSNA, Abemaciclib, Breast cancer, Lymph node metastasis, Sentinel lymph node

## Abstract

**Purpose:**

To validate the predictive value of Total Tumor Load (TTL), based on Cytokeratin 19 (CK19) mRNA copy number measured by OSNA in sentinel lymph nodes (SLNs), and to establish an optimal cut-off for identifying HR + /HER2– breast cancer (BC) patients at high risk of ≥ 4 metastatic lymph nodes (LNs), eligible for adjuvant abemaciclib therapy.

**Patients/Methods:**

A retrospective, international, multicenter study pooling data from six studies conducted between 2000 and 2016 in adult BC patients with SLN biopsies analyzed by OSNA. Variables were TTL (CK19 mRNA copies/µL) in SLNs, number of LNs removed, and number of metastatic ones. We constructed receiver operating characteristics (ROC) curves to determine optimal TTL values and multivariate logistic regression models to identify relevant prognostic factors for predicting ≥ 4 LN metastases.

**Results:**

We included 3804 patients with HR + /HER2- tumors of any histological type, complete axillary LN dissection, and > 4 LNs removed; 608 (16.0%) had ≥ 4 metastatic LNs and 439 had lobular BC. At cut-offs of 99,500 and 101,000 copies/µL, TTL had a 32.3% and 32.4% PPV. In lobular BC, cut-offs between 92,305 and 108,700 copies/µL reached a 40% PPV. In lobular HR + /HER2- BC, a multivariate model including tumor grade, TTL, and lymphovascular invasion reached a 49% PPV for predicting ≥ 4 metastatic LNs, yielding a nomogram and an online calculator for use in routine practice.

**Conclusion:**

TTL measured using OSNA predicts ≥ 4 metastatic LNs in patients with luminal-like BC, with a proposed optimal threshold of 100,000 copies/µL.

**Trial registration:**

Not applicable.

**Supplementary Information:**

The online version contains supplementary material available at https://doi.org/10.1007/s12094-026-04426-1.

## Background

Breast cancer (BC) is the most common type of cancer in women and the leading cancer-related cause of death. Hormone receptor positive/human epidermal growth factor receptor 2 negative (HR +/HER2-) BC is the most common subtype, accounting for 50 to 60% of all cases, and is generally associated with a favorable prognosis [1]. However, a subset of patients may experience disease recurrence several years after initial treatment [2].

In particular, node-positive early BC patients are at high risk of recurrence (up to 30% at 5 years) [3]. To optimize the treatment choice for these patients, new therapies targeting the cyclin-dependent kinase 4/6 (CDK4/6) have emerged in combination with ET [3]. Abemaciclib, a CDK4/6 inhibitor, is approved as adjuvant therapy for HR +/HER2- early BC patients at high risk of recurrence, based on the phase III monarchE trial [4]. In this trial, adding abemaciclib to ET significantly reduced early recurrence and metastasis in patients with ≥ 4 positive LNs or 1–3 LNs plus grade 3 and/or tumors > 5 cm [5], and improved overall survival compared to ET alone after two years [6].

Identification of high-risk patients who may benefit from abemaciclib treatment requires analysis of several LNs. While complete axillary lymph node dissection (ALND) has been the standard practice in patients with positive sentinel LN (SLN), current guidelines recommend a de-escalation of the surgical approach, consisting of targeted axillary dissection in patients achieving good response to neoadjuvant therapy, or ALND omission in selected patient groups based on clinical-pathological variables [7, 8]. This de-escalation aims to prevent overtreatment and morbidity associated with ALND but precludes the identification of patients who may benefit from abemaciclib treatment [7]. Conversely, surgical escalation is not recommended upon detection of one or two positive SLNs to determine eligibility for abemaciclib treatment [9, 10]. In this scenario, several models for predicting ≥4 positive LNs were developed [11], based on traditional clinical and pathological variables associated with recurrence, such as LN involvement, tumor size, histological grade and type, and lymphovascular invasion [11, 12].

One-step nucleic acid amplification (OSNA) directly measures cytokeratin-19 (CK19) mRNA levels in fresh SLNs, enabling objective and accurate detection of metastasis, as CK19 is expressed in epithelial cells but absent in normal lymphoid tissue and present in over 95% of breast carcinomas. This assay allows calculation of the tumor load (TL), defined as the total number of mRNA copies in each positive SLN (copies/µL). The Total Tumor Load (TTL), calculated as the sum of the TL in all positive SLNs, is a valuable predictive tool for guiding decisions regarding ALND and for predicting prognosis of non-SLN metastasis and risk of recurrence [13–15]. Moreover, TL and TTL have been explored to predict the presence of four or more metastatic LNs, with established mRNA copy thresholds of > 100,000 for TL and > 110,000 for TTL predictive of ≥4 LN metastasis [16, 17].

Despite the promising results of these studies, the number of patients assessed was small, and larger studies are needed to evaluate the value of CK19 mRNA copy number to predict ≥4 LN metastasis. This retrospective study aimed to confirm the capacity of OSNA to predict ≥ 4 metastases in a large patient series and to determine the best cut-off value of TTL from SLN analyzed by OSNA for predicting ≥ 4 metastatic LNs in patients with HR +/HER2- BC of any histological type using pooled data from six previous studies. The secondary objectives were to assess cut-off value accuracy in the subgroup of patients with lobular carcinoma and to develop logistic regression models that include other variables associated with recurrence to improve accuracy.

## Methods

### Study design, population, and data source

This was a retrospective, international, multicenter study in adult patients (≥18 years) with a BC diagnosis of any histological type. This evaluation study used pooled data from six previous studies conducted between 2000 and 2016 in five hospitals in Spain, Italy, and Portugal, where OSNA was routinely performed: SOLO 1113 [13], Piñero-Madrona 2014 [18], DiFilippo 2015 [19], PLUTTO [14], PLUTTO Validation [20], and PORTTLE [15]. These studies included HR +/HER2- breast cancer patients with SLN biopsy (SLNB) analyzed by OSNA and complete ALND with ≥ 4 lymph nodes examined by routine H&E (n = 3804). Patients with carcinoma in situ, another metastatic tumor, bilateral BC, or prior chemotherapy or hormonal therapy were excluded.

Patients provided their written informed consent to participate in the previous studies. This study was conducted following the principles of the Helsinki Declaration (2024 Fortaleza, Brazil) [21]. The study protocol for this evaluation of previously collected data was approved by the local Ethics Committee at each of the participating sites (Hospital Vall d’Hebron, Virgen de la Arrixaca University Hospital, Regina Elena National Cancer Institute, and Centro Hospitalar e Universitário São João).

### Outcomes and variables

The primary objective was to validate the previously reported TTL cut-off of 100,000 copies in SLNs analyzed by OSNA for predicting ≥ 4 metastatic lymph nodes (SLNs and non-SLNs) in HR +/HER2- breast cancer patients, considering node counts and HR/HER2 status according to ASCO/CAP guidelines [22].

For the secondary objectives, we considered basic demographic data and primary tumor data, including maximum tumor diameter (mm), histological type of BC according to the WHO classification [23], tumor grade, lymphovascular invasion, and percentage of Ki67-positive cells.

### Statistical analysis

Qualitative variables are presented as frequencies and percentages, and quantitative variables are presented as the mean and standard deviation (SD).

Before data pooling, pairwise comparisons between the six studies were performed using Fisher’s exact test and the Chi-square test for categorical variables and Mann–Whitney test and independent t-test for continuous variables.

To determine the best TTL cut-off value for predicting the presence of four or more metastatic LNs, we constructed receiver operating characteristic (ROC) curves and calculated the area under the curve (AUC) and the Youden’s J index. Accuracy, discordance, sensitivity, specificity, false positives, false negatives, positive predictive value (PPV), negative predictive value (NPV), and kappa index are presented for selected TTL cut-off values. Corresponding 95% confidence intervals (95% CIs) were calculated where applicable. We analyzed TTL thresholds ranging from 100 to 39,020,300,000.0 copies/µL, and presented the results for the previously reported 100,000 copies/µL [16]. Although higher TTL thresholds may likely improve test performance, they would identify very few patients as positive. Therefore, an optimal TTL threshold should balance adequate ROC parameters with the need to minimize missed cases. Of all the parameters derived from the ROC analysis, we focused on optimizing the PPV, which measures the proportion of true positives among patients with positive results. Unlike sensitivity and specificity, which reflect the intrinsic ability of a test to identify positive and negative patients, PPV provides an intuitive and clinically meaningful measure, with a direct application in routine clinical practice to guide treatment decisions.

For the secondary objectives, we performed the same analyses in the subgroup of patients with lobular HR +/HER2- BC. Moreover, we constructed logistic regression models using univariate and multivariate analyses to identify relevant prognostic factors for predicting the presence of ≥ 4 metastatic LNs and refine the predictive value of the TTL and a nomogram yielding a probability score was constructed with the selected predictive factors.

Calibration of the multivariable lobular model was assessed using the Hosmer–Lemeshow goodness-of-fit test with 10 groups and the scaled Brier Score.

Statistical significance was set at a two-sided α = 0.05 (p < 0.05). All statistical analyses were performed using R software, version 4.5.1, R Foundation for Statistical Computing (Vienna, Austria).

## Results

### Characteristics of study patients

Of 9456 breast cancer patients initially identified, 3804 met all eligibility criteria, including SLNB analyzed by OSNA, ALND with ≥ 4 lymph nodes examined, and HR +/HER2- tumors. The most frequent histological type was invasive carcinoma of no special type (74%), while 12% had lobular carcinoma; over half of the patients had histological grade 2 tumors, and 38% presented with lymphovascular invasion (Table 1). A total of 6863 SLNs and 51,539 non-sentinel LNs were removed, of which 4934 and 4169 were metastatic, respectively. At least four metastatic non-sentinel LNs were detected in 308 patients (8%). Of them, 608 (16.0%) had ≥ 4 metastatic LNs (sentinel and non-sentinel).

**Table 1:** Characteristics of patients, primary tumors, and dissected lymph nodes. N = 3804

Demographic characteristics	
Age (years), mean (SD) n = 3788	57 (12)
Sex, n (%) n = 303	
Female, n (%)	299 (99)
Male, n (%)	4 (1)
Primary tumor characteristics	
Primary tumor size (maximum diameter), n = 3778	
Maximum diameter (mm), *mean (SD)*	20 (12)
≤ 2 cm, n (%)	37 (1)
> 2 cm y ≤ 5 cm, n (%)	62 (2)
> 5 cm, n (%)	3679 (97)
Histological type, n (%) n = 3762	
Invasive carcinoma of no special type	2793 (74)
Other	530 (14)
Lobular carcinoma	439 (12)
Tumor grade, n (%) n = 3629	
1	781 (22)
2	2157 (59)
3	691 (19)
Lymphovascular invasion, n (%) n = 3461	
No	2,153 (6)
Yes	1308 (38)
Estrogen receptors +, n (%) n = 3780	
Negative	23 (1)
Positive	3757 (99)
Progesterone receptors +, n (%) n = 3777	
Negative	414 (11)
Positive	3363 (89)
HER2 +, n (%) n = 3804	
Negative	3804 (100)
Positive	0 (0)
HR +/HER2- (luminal), n (%) n = 3804	
No	0 (0)
Yes	3804 (100)
Ki67 (%), n = 2101	
Mean (SD)	17 (15)
Ki67-positive cells ≥ 20%, n (%)	721 (34)
Sentinel LN dissection	
Sentinel LNs removed, n (%) n = 3804	6863
Mean (SD) per patient	2 (1)
1	1781 (47)
2	1273 (33)
3	516 (14)
≥ 4	234 (6)
Metastatic sentinel LNs, n (%) n = 3804	4934
Mean (SD) per patient	1 (1)
0	120 (3%)
1	2699 (71%)
2	767 (20%)
3	175 (5%)
≥ 4	43 (1%)
Axillary lymphadenectomy	
Non-sentinel LNs removed, n (%) n = 3804	51,539
Mean (SD) per patient	15 (7)
Metastatic non-sentinel LNs, n (%) n = 3804	4,169
Mean (SD) per patient	1 (3)
0	2522 (66)
1	583 (15)
2	234 (6)
3	157 (4)
4	81 (2)
5–6	81 (2)
≥ 7	146 (4%)

*HR* hormone receptor, *LN* lymph node, *SD* standard deviation

Comparison of data from the HR +/HER2- population for all variables across studies showed no relevant differences between study populations, indicating that the data from the six studies were homogeneous, thus allowing pooled analysis. Table S1 summarizes comparisons for the primary variable (i.e., presence of ≥ 4 metastatic LNs).

### Predictive value of TTL cut-off points

The ROC curve analyses showed that at thresholds of 99,500 and 101,000 copies/µL, TTL had a PPV of 32.3 and 32.4% with 0.3425 and 0.3401 Youden indices, respectively. The AUC for the ROC analysis was 74.06. This result indicates that, at this threshold, TTL would predict four or more metastases in 32% of patients (Table 2). While PPV values increased with TTL thresholds, more patients with ≥ 4 metastatic LNs would be missed and very few patients would remain as candidates to receive abemaciclib, given that patients with higher TTLs (> 100,000 copies/µL) are uncommon (Table S2). Therefore, a 100,000 copies/µL threshold was considered the best to predict ≥ 4 LN metastasis.

**Table 2:** ROC curve analysis of selected total tumor load (TTL) thresholds for predicting ≥ 4 metastatic lymph nodes in patients with HR +/HER2- breast cancer

TTL threshold	Specificity	Sensitivity	Accuracy	TN	TP	FN	FP	PPV	NPV	Youden
94,705	77.1	57.7	74.0	2465	351	257	731	32.4	90.6	0.3486
97,000	77.3	56.9	74.0	2470	346	262	726	32.3	90.4	0.3419
99,500	77.3	56.9	74.1	2472	346	262	724	32.3	90.4	0.3425
101,000	77.6	56.4	74.2	2480	343	265	716	32.4	90.3	0.3401
102,400	77.7	56.4	74.3	2483	343	265	713	32.5	90.4	0.3411
103,600	77.8	56.4	74.4	2488	343	265	708	32.6	90.4	0.3426
104,500	77.9	55.9	74.4	2490	340	268	706	32.5	90.3	0.3383
105,850	77.9	55.8	74.4	2490	339	269	706	32.4	90.3	0.3367
106,850	77.9	55.8	74.4	2491	339	269	705	32.5	90.3	0.3370
107,550	78	55.8	74.5	2494	339	269	702	32.6	90.3	0.3379
108,400	78	55.6	74.4	2494	338	270	702	32.5	90.2	0.3363
109,550	78	55.4	74.4	2494	337	271	702	32.4	90.2	0.3346
110,455	78.7	55.3	74.9	2515	336	272	681	33.0	90.2	0.3396

*FN* false negatives, *FP* false positives, *NPV* negative predictive value, *PPV* positive predictive value, *ROC* receiver operating characteristic, *TN* true negatives, *TP* true positives, *TTL* total tumor load

### Predictive value of TTL cut-off points in patients with HR + HER- lobular tumors

Of the 3804 evaluable patients with HR +/HER2- breast tumors, 439 were lobular. In this subpopulation, at a TTL threshold between 92,305 and 108,700 copies/µL, the PPV reached 40% with a Youden index > 0.39 (Table 3). The AUC for the ROC analysis was 76.5. While PPV values increased with TTL thresholds, few patients with ≥ 4 metastatic LNs would be identified and potential candidates to receive abemaciclib would be missed (Table S3).

**Table 3:** ROC curve analysis of selected total tumor load (TTL) thresholds for predicting ≥ 4 metastatic lymph nodes in patients with lobular HR +/HER2- breast cancer

TTL threshold	Specificity	Sensitivity	Accuracy	TN	TP	FN	FP	PPV	NPV	Youden
88,155	76.1	64.8	73.8	267	57	31	84	40.4	89.6	0.4084
92,305	76.1	63.6	73.6	267	56	32	84	40.0	89.3	0.3970
98,500	76.1	62.5	73.3	267	55	33	84	39.6	89.0	0.3857
102,500	76.4	62.5	73.6	268	55	33	83	39.9	89.0	0.3885
103,100	76.6	62.5	73.8	269	55	33	82	40.1	89.1	0.3914
105,300	76.9	62.5	74.0	270	55	33	81	40.4	89.1	0.3942
108,700	77.5	62.5	74.5	272	55	33	79	41.0	89.2	0.3999
110,145	77.8	62.5	74.7	273	55	33	78	41.4	89.2	0.4028
115,145	78.1	62.5	74.9	274	55	33	77	41.7	89.3	0.4056

*FN* false negatives, *FP* false positives, *NPV* negative predictive value, *PPV* positive predictive value, *ROC* receiver operating characteristic, *TN* true negatives, *TP* true positives, *TTL* total tumor load

### Multivariate predictive models

The univariate logistic regression analysis yielded significant differences in the number of non-SLNs removed, tumor grade, maximum tumor diameter, lymphovascular invasion, and TTL for predicting ≥ 4 metastatic LNs in patients with HR +/HER2- BC (Table S4). In a multivariate logistic regression model including these significant variables, they remained statistically significant. However, the multivariate model did not improve the PPV of TTL in the ROC curve analysis (Table S5).

In the subpopulation with lobular HR +/HER2- BC, the univariate analyses showed that increased tumor grade, increased TTL, and lymphovascular invasion significantly increased the odds ratio (OR) for presenting ≥4 metastatic LNs (Table 4).

**Table 4:** Univariate and multivariate logistic regression models for predicting the presence of ≥ 4 metastatic lymph nodes in patients with lobular HR +/HER2- breast cancer, n = 332

		Univariate model				Multivariate model			
Variable	Levels	OR	Lower 95% CI	Upper 95% CI	P-Value	OR	Lower 95% CI	Upper 95% CI	P-Value
Ki67 (%)	> = 20 vs < 20	0.93	0.46	1.8	0.8257				
Progesterone receptors	Positive vs Negative	1.34	0.66	3.03	0.4464				
Age		1.01	0.99	1.03	0.2295				
Maximum tumor diameter (mm)		1.02	1.01	1.04	0.0005				
Number of non-sentinel nodes removed		1.06	1.03	1.1	0.0003				
Tumor grade (num)		2.39	1.44	4.05	0.001	1.84	1.05	3.32	0.0369
Lymphovascular invasion	Yes vs No	3.47	2.05	5.86	< 0.0001	2.25	1.22	4.11	0.0086
Total tumor load (log_10_)		1.42	1.3	1.58	< 0.0001	1.38	1.24	1.54	< 0.0001

*CI* confidence interval, *OR* odds ratio

**Fig. 1:**
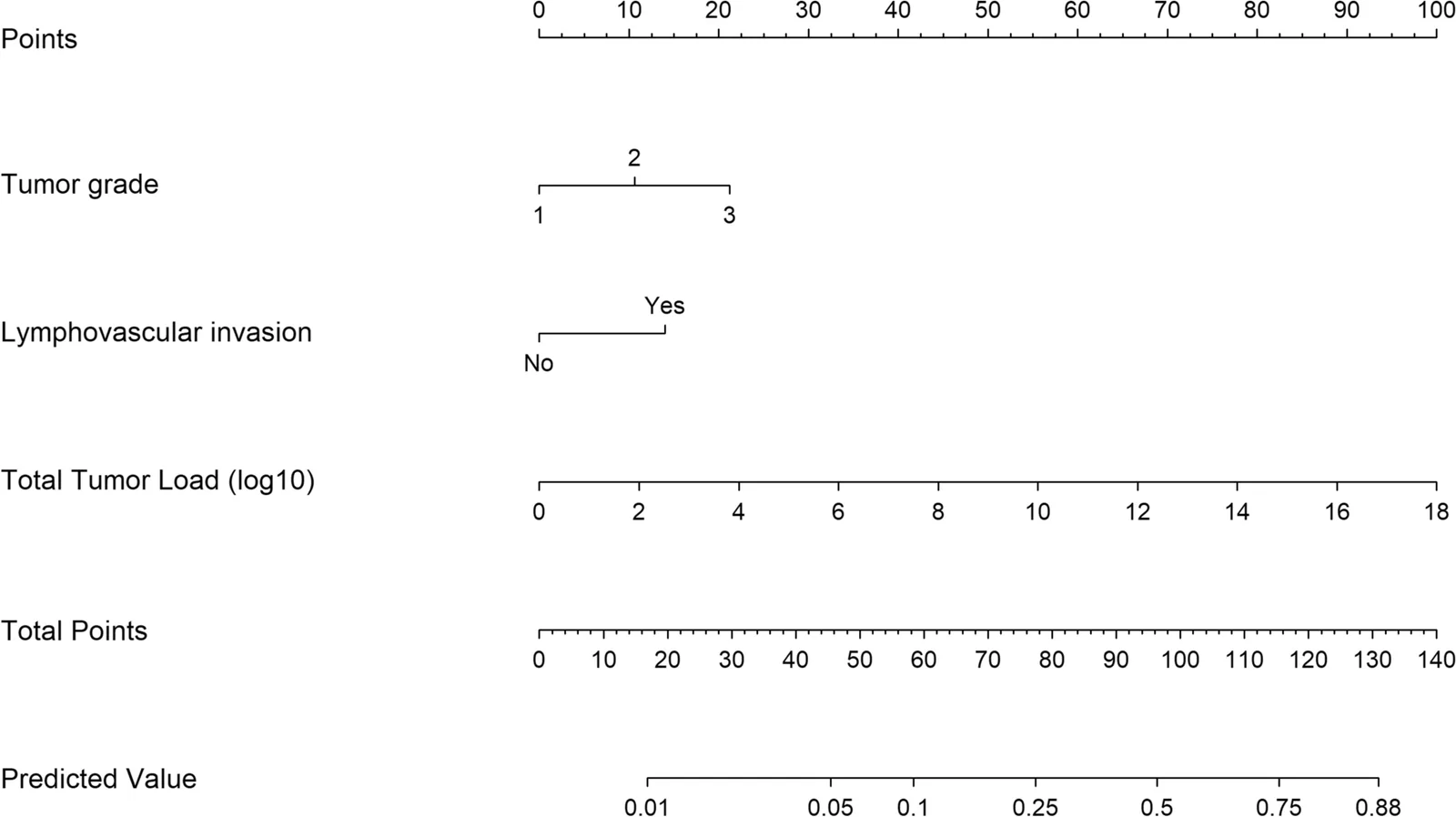
Proposed nomogram for predicting ≥ 4 metastatic sentinel and non-sentinel lymph nodes in patients with HR +/HER2- lobular breast carcinoma

These variables remained significant in a multivariate model. ROC analysis of the multivariate model thresholds showed an improved predictive capacity compared to TTL alone, reaching a PPV of 49% (Table 5). Based on these observations, we developed a nomogram for the application of these parameters (tumor grade, lymphovascular invasion, and TTL) to predict the presence of ≥4 metastatic LNs in patients with lobular carcinoma (Fig. 1). Based on the nomogram, we developed a calculator that will be available to clinicians through the website of the Spanish Society of Senology and Breast Pathology (*Sociedad Española de Senología y Patología Mamaria*, SESPM) to determine patients' recurrence risk and guide treatment decisions.

**Table 5:** ROC curve analysis of the multivariate model for predicting ≥ 4 metastatic lymph nodes in patients with lobular HR +/HER2- breast cancer

Threshold	Specificity	Sensitivity	Accuracy	TN	TP	FN	FP	PPV	NPV	Youden
0.265470861	78.3	70.3	76.5	202	52	22	56	48.1	90.2	0.4856
0.271646283	78.7	70.3	76.8	203	52	22	55	48.6	90.2	0.4895
0.279005422	79.1	68.9	76.8	204	51	23	54	48.6	89.9	0.4799
0.284900831	79.1	67.6	76.5	204	50	24	54	48.1	89.5	0.4664
0.287391074	79.5	67.6	76.8	205	50	24	53	48.5	89.5	0.4702
0.288916794	79.8	67.6	77.1	206	50	24	52	49.0	89.6	0.4741
0.294493695	79.8	66.2	76.8	206	49	25	52	48.5	89.2	0.4606

*FN* false negatives, *FP* false positives, *NPV* negative predictive value, *PPV* positive predictive value, *ROC* receiver operating characteristic, *TN* true negatives, *TP* true positives

## Discussion

This retrospective, multicenter study evaluating a large dataset from patients with HR +/HER2- BC of any histological type showed that the TTL of CK19 mRNA in SLNs is a valuable predictor of the presence of four or more metastatic LNs. At the previously established cut-off values of ⁓100,000 copies/µL, TTL had a PPV between 32.3 and 32.4%, indicating that almost one-third of patients with TTL ≥100,000 copies/µL present four or more metastatic LNs. In the subpopulation of patients with lobular carcinoma, the TTL's PPV at this cut-off reached 40%, reflecting improved predictive capacity in this BC subtype. Multivariate models including factors associated with BC recurrence in addition to TTL did not show improved performance in predicting ≥4 LN metastasis. However, in the subpopulation of patients with HR +/HER2- lobular carcinoma, a multivariate model including TTL, tumor grade, and lymphovascular invasion reached a 49% PPV, indicating that these parameters predicted ≥4 LN metastasis in almost half of the cases.

Our results regarding the performance of TTL for predicting ≥4 LN metastasis in the overall population of patients with HR +/HER2- (luminal) BC align with previous studies in smaller populations [16, 17]. Kenny et al*.* analyzed 134 patients and found that TTL values ≥110,000 copies/µL in the SLN correlated with ≥4 LN metastasis, and established this value as the optimal cut-off point in ROC analysis [16]. Similarly, the study by Ohi et al*.* reported that the CK19 copy number correlated with ≥4 LN metastasis in the 130 patients included, and established that at a 100,000 copies/µL threshold, the frequency of patients with ≥4 LN metastasis was 35.3%, in line with our study showing a 32% PPV [17]. The results from this and previous studies showed minor discrepancies, likely due to differences in the clinical and pathological characteristics of the study populations. Moreover, these previous studies were small and had variable representation of patients with ≥ 4 LN metastases, ranging between 18.5 and 25.4% [16, 17], whereas in our larger study 16.0% of patients had ≥ 4 LN metastases. Nevertheless, the optimal thresholds and predictive performances were similar across studies, supporting TTL in SLNs measured by OSNA as a reliable tool for predicting ≥ 4 LN metastases.

The need to perform ALND in patients with positive SLNs has long been a topic of debate, despite the importance of detecting positive axillary LNs for disease staging and assessing patients’ recurrence risk to decide on the most appropriate adjuvant treatment [24]. To address this need, models for predicting ≥ 4 LN metastases, which defines a high recurrence risk and therefore indication to receive abemaciclib, have been developed [11]. At the predicted probability threshold of 5%, the nomogram developed by Yang et al. had a PPV of 27.8% and a sensitivity of 94.8% [11, 25]. Some of these factors, such as LN involvement and tumor histology, have traditionally been assessed using methods that depend on the observer's interpretation and may therefore be subjective. The histological assessment of positive SLNs may be subject to sample bias, since only a fragment of the SLN tissue is examined, and may be influenced by intra- and interobserver variability, as well as expertise. Given these limitations, OSNA is a direct, objective, and quantifiable method of measuring the total amount of CK19 mRNA in the entire SLN. Importantly, in the context of evolving clinical practice, where axillary lymph node dissection is increasingly omitted, approaches incorporating OSNA-derived measures such as TTL may provide a practical alternative to estimate axillary involvement and support treatment decision-making. Although previous models showed that clinical and pathological characteristics are strong predictors of LN metastasis [11], incorporating OSNA into multivariate models has been shown to optimize performance.

This study analyzed pooled data from six studies, including a total of 3804 patients with HR +/HER2- (luminal) carcinoma. Of these patients, 439 had lobular carcinoma, enabling a subgroup analysis of this BC subtype to be performed. This BC type is typically underrepresented in smaller studies due to its lower incidence (5–15%) [26]. The TTL cut-off point chosen for this study to predict the presence of ≥4 LN metastases performed particularly well in this subgroup of patients, with a PPV of 40% for TTL alone and 49% for the multivariate model. Unlike other types of BC, lobular BC cells are discohesive due to loss of E-cadherin, infiltrating as single-cell strands that are hard to detect on mammography [26]. They also spread diffusely to lymph nodes, causing subtle morphological changes that complicate accurate clinical staging [27]. Moreover, detecting metastases in SLNs using histological methods is challenging, because they often develop as single cells or small foci [27]. Consequently, the use of OSNA and the optimal performance of TTL in predicting ≥4 LN metastases in half of patients with lobular HR +/HER2- BC are particularly valuable.

Unlike sensitivity and specificity, which refer to the intrinsic ability of a test to identify positive and negative patients, PPV refers to the proportion of patients who are identified correctly, reflecting the test’s performance in the real world [28]. Although it should be interpreted with caution, as it largely depends on the prevalence of the condition in the population, PPV is the preferred measure for clinicians [28]. In the case of TTL cut-offs, PPV increased as TTL values increased, thereby improving the PPV. However, fewer patients would be identified as positive, given the low frequency of patients with high TTL, decreasing the clinical utility of the diagnostic test. Nevertheless, oncologists should base treatment decisions on additional clinical and pathological characteristics, particularly for treatments such as abemaciclib, which is associated with adverse events. Given the current recommendations regarding the omission of ALND, TTL emerges as a valuable tool for BC staging and for identifying candidate patient to receive abemaciclib treatment. Moreover, based on the results of two clinical trials, the axillary surgical approach is evolving [8, 29], and SLNB is no longer recommended in all cases. In our cohort of 3804 patients, the analysis and positive assessment of one SLN would confirm the eligibility of 513 patients (13.5%) with grade 3 tumors, 481 patients (12,6%) with Ki67 > 20%, and 74 patients (1,94%) with a tumor ≥ 50 mm to receive treatment with abemaciclib. These observations support the importance of performing SLNB for BC staging and to guide treatment decisions.

The study’s limitations include its retrospective design and the use of pooled data from multiple studies, leading to some missing and heterogeneous variables. Despite this, the results confirm that a TTL cut-off of 100,000 copies/µL reliably predicts ≥ 4 metastatic LNs in real-world practice.

## Conclusions

In this large cohort, TTL measured in SLNs by OSNA reliably predicts the presence of ≥ 4 metastatic LNs in HR +/HER2- breast cancer patients with an optimal threshold of 100,000 CK19 mRNA copies/µL. Combined with clinical-pathologic factors, TTL identifies high-risk patients and helps guide selection of adjuvant therapy such as abemaciclib, particularly in the context of de-escalated axillary surgery.

## Supplementary Information

Below is the link to the electronic supplementary material.

Supplementary file1 (DOCX 52 KB)

## Data Availability

The datasets generated and/or analyzed during the current study are available from the corresponding author on reasonable request.

## References

[1] Yersal O, Barutca S. Biological subtypes of breast cancer: prognostic and therapeutic implications. World J Clin Oncol. 2014;5:412–24. 10.5306/wjco.v5.i3.412. PMC4127612 25114856

[2] Pan H, Gray R, Braybrooke J, Davies C, Taylor C, McGale P, et al. 20-Year risks of breast-cancer recurrence after stopping endocrine therapy at 5 years. N Engl J Med. 2017;377:1836–46. 10.1056/NEJMoa1701830. PMC5734609 29117498

[3] Loibl S, André F, Bachelot T, Barrios CH, Bergh J, Burstein HJ, et al. Early breast cancer: ESMO clinical practice guideline for diagnosis, treatment and follow-up. Ann Oncol. 2024;35:159–82. 10.1016/j.annonc.2023.11.016. 38101773

[4] Rastogi P, O’Shaughnessy J, Martin M, Boyle F, Cortes J, Rugo HS, et al. Adjuvant Abemaciclib plus endocrine therapy for hormone receptor–positive, human epidermal growth factor receptor 2–negative, high-risk early breast cancer: results from a preplanned monarchE overall survival interim analysis, including 5-Year efficacy outcomes. J Clin Oncol. 2024;42:987–93. 10.1200/JCO.23.01994. PMC10950161 38194616

[5] Johnston SRD, Harbeck N, Hegg R, Toi M, Martin M, Shao ZM, et al. Abemaciclib combined with endocrine therapy for the adjuvant treatment of HR+, HER2−, node-positive, high-risk, early breast cancer (monarchE). J Clin Oncol. 2020;38:3987–98. 10.1200/JCO.20.02514. PMC7768339 32954927

[6] Czapar M. Lilly’s Verzenio^®^ (abemaciclib) increases overall survival in HR+, HER2-, high-risk early breast cancer with two years of therapy. 2025

[7] de BJ, Tvedskov TF, Rydén L, Szulkin R, Reimer T, Kühn T, et al. Omitting axillary dissection in breast cancer with sentinel-node metastases. N Engl J Med. 2024;390:1163–75. 10.1056/NEJMoa2313487. (**Massachusetts Medical Society;**). 38598571

[8] Gentilini OD, Botteri E, Sangalli C, Galimberti V, Porpiglia M, Agresti R, et al. Sentinel lymph node biopsy vs no axillary surgery in patients with small breast cancer and negative results on ultrasonography of axillary lymph nodes: the SOUND randomized clinical trial. JAMA Oncol. 2023;9:1557–64. 10.1001/jamaoncol.2023.3759. PMC10514873 37733364

[9] Williams AD, Ruth K, Shaikh SS, Vasigh M, Pronovost M, Aggon AA, et al. Should patients with HR+/HER2− breast cancer and 1–2 positive sentinel nodes undergo axillary dissection to determine candidacy for adjuvant abemaciclib? Cancer. 2024;130:1052–60. 10.1002/cncr.35136. PMC11422773 38018862

[10] Jankowski C, Houvenaeghel G, Renaudeau C, Leveque J, Marchal F, Benbara A, et al. How can we optimize the surgical management of the axilla in breast cancer since the MonarchE trial? Breast Cancer Res Treat. 2025;212:13–21. 10.1007/s10549-025-07642-5. 40329152

[11] Yang Z, Lan X, Huang Z, Yang Y, Tang Y, Jing H, et al. Development and external validation of a nomogram to predict four or more positive nodes in breast cancer patients with one to three positive sentinel lymph nodes. Breast. 2020;53:143–51. 10.1016/j.breast.2020.08.001. PMC7451418 32823167

[12] Zambelli A, Gallerani E, Garrone O, Pedersini R, Rota Caremoli E, Sagrada P, et al. Working tables on hormone receptor positive (HR+), human epidermal growth factor receptor 2 negative (HER2-) early stage breast cancer: defining high risk of recurrence. Crit Rev Oncol Hematol. 2023;191:104104. 10.1016/j.critrevonc.2023.104104. 37659765

[13] Peg V, Espinosa-Bravo M, Vieites B, Vilardell F, Antúnez JR, de Salas MS, et al. Intraoperative molecular analysis of total tumor load in sentinel lymph node: a new predictor of axillary status in early breast cancer patients. Breast Cancer Res Treat. 2013;139:87–93. 10.1007/s10549-013-2524-z. 23576079

[14] Peg V, Sansano I, Vieites B, Bernet L, Cano R, Córdoba A, et al. Role of total tumour load of sentinel lymph node on survival in early breast cancer patients. Breast. 2017;33:8–13. 10.1016/j.breast.2017.02.011. 28254641

[15] Fougo JL, Amendoeira I, Brito MJ, Correia AP, Gonçalves A, Honavar M, et al. Sentinel node total tumour load as a predictive factor for non-sentinel node status in early breast cancer patients—The porttle study. Surg Oncol. 2020;32:108–14. 10.1016/j.suronc.2019.11.008. 31987574

[16] Kenny R, Wong G, Gould L, Odofin O, Bowyer R, Sotheran W. Can one-step nucleic acid amplification assay predict four or more positive axillary lymph node involvement in breast cancer patients: a single-centre retrospective study. Ann R Coll Surg Engl. 2022;104:216–20. 10.1308/rcsann.2021.0154. PMC10334894 34928727

[17] Ohi Y, Umekita Y, Sagara Y, Rai Y, Yotsumoto D, Matsukata A, et al. Whole sentinel lymph node analysis by a molecular assay predicts axillary node status in breast cancer. Br J Cancer. 2012;107:1239–43. 10.1038/bjc.2012.387. PMC3494436 22929881

[18] Piñero-Madrona A, Ruiz-Merino G, Bernet L, Miguel-Martínez B, Vicente-García F, Viguri-Díaz MA, et al. Tumoral load quantification of positive sentinel lymph nodes in breast cancer to predict more than two involved nodes. Breast. 2014;23:859–64. 10.1016/j.breast.2014.09.005. 25283682

[19] Di Filippo F, Giannarelli D, Bouteille C, Bernet L, Cano R, Cunnick G, et al. Elaboration of a nomogram to predict non sentinel node status in breast cancer patients with positive sentinel node, intra-operatively assessed with one step nucleic acid amplification method. J Exp Clin Cancer Res. 2015;34:136. 10.1186/s13046-015-0246-2. PMC4632276 26538019

[20] Piñero-Madrona A, Ripoll-Orts F, Sánchez-Méndez JI, Chaves-Benito A, Gómez-de la Bárcena MR, Calatrava-Fons A, et al. External validation of a prognostic model based on total tumor load of sentinel lymph node for early breast cancer patients. Breast Cancer Res Treat. 2020;181:339–45. 10.1007/s10549-020-05623-4. PMC7188708 32253684

[21] WMA - The World Medical Association-Declaration of Helsinki [Internet]. [cited 2025 Sept 10]. https://www.wma.net/what-we-do/medical-ethics/declaration-of-helsinki/. Accessed 10 Sept 2025

[22] Allison KH, Hammond MEH, Dowsett M, McKernin SE, Carey LA, Fitzgibbons PL, et al. Estrogen and progesterone receptor testing in breast cancer: ASCO/CAP guideline update. J Clin Oncol. 2020;38:1346–66. 10.1200/JCO.19.02309. 31928404

[23] WHO Classification of Tumours Editorial Board. Breast Tumours [Internet]. 2019 [cited 2025 Nov 11]. https://publications.iarc.who.int/Book-And-Report-Series/Who-Classification-Of-Tumours/Breast-Tumours-2019. Accessed 11 Nov 2025

[24] Rose FD, Meduri B, Santis MCD, Ferro A, Marino L, Colciago RR, et al. Rethinking breast cancer follow-up based on individual risk and recurrence management. Cancer Treat Rev. 2022. 10.1016/j.ctrv.2022.102434. (**Elsevier;**). 35933845

[25] Unal B, Gur AS, Beriwal S, Tang G, Johnson R, Ahrendt G, et al. Predicting likelihood of having four or more positive nodes in patient with sentinel lymph node-positive breast cancer: a nomogram validation study. Int J Radiat Oncol Biol Phys. 2009;75:1035–40. 10.1016/j.ijrobp.2008.12.028. 19327916

[26] Wilson N, Ironside A, Diana A, Oikonomidou O. Lobular breast cancer: a review. Front Oncol. 2021. 10.3389/fonc.2020.591399. (**Frontiers;**). PMC7844138 33520704

[27] Fernández B, Paish EC, Green AR, Lee AHS, Macmillan RD, Ellis IO, et al. Lymph-node metastases in invasive lobular carcinoma are different from those in ductal carcinoma of the breast. J Clin Pathol. 2011;64:995–1000. 10.1136/jclinpath-2011-200151. 21712309

[28] Labrique AB, Pan WK-Y. Diagnostic tests: understanding results, assessing utility, and predicting performance. Am J Ophthalmol. 2010;149:878-881.e2. 10.1016/j.ajo.2010.01.001. PMC6359896 20510686

[29] Reimer T, Stachs A, Veselinovic K, Kühn T, Heil J, Polata S, et al. Axillary surgery in breast cancer—primary results of the INSEMA trial. N Engl J Med. 2025;392:1051–64. 10.1056/NEJMoa2412063. 39665649

