# Supplementary material for: Evaluating total tumor load (TTL) to predict metastasis in four or more axillary lymph nodes in HR +/HER2- breast cancer patients: the TULIP4N + study
Source: Clin Transl Oncol. 2026 Jun 6;28(11):5231–9. doi: 10.1007/s12094-026-04426-1 (PMC13694496; doi:10.1007/s12094-026-04426-1)
Supplement: Supplementary file 1 [file 12094_2026_4426_MOESM1_ESM.docx]

# Supplementary Materials for the Submission “Evaluating Total Tumor Load (TTL) to Predict Metastasis in Four or More Axillary Lymph Nodes in HR+/HER2- Breast Cancer Patients: the TULIP4N+ Study” by Zamora *et al*.

**Journal:** Clinical Translational Oncology

**Authors:** Esther Zamora, Antonio Piñero-Madrona, Isabella Sperduti, Anna Sapino, José Luis Fougo, Vicente Peg

**Corresponding author:**

Dr. Vicente Peg

ORCiD ID: 0000-0002-5203-6166

email: vicente.peg@vallhebron.cat

# Supplementary Tables

| **Table S1.** Comparison of frequencies of patients with four or more metastatic non-sentinel lymph nodes across the six studies included in this pooled analysis (HR+/HER2- population). N=3830 | | | | | | |
| --- | --- | --- | --- | --- | --- | --- |
|  | **DiFilippo + Validation^a^** | **Piñero-Madrona^b^** | **PLUTTO^c^** | **PLUTTO Validation^d^** | **PORTTLE^e^** | **SOLO-STUDY^f^** |
| N | 1946 | 455 | 303 | 25 | 529 | 572 |
| **≥4 metastatic non-sentinel nodes, *n (%)*** | 157 (8.07) | 34 (7.47) | 30 (9.90) | 5 (20.00) | 45 (8.51) | 42 (7.34) |
| *P*-value pairwise comparisons | |  |  |  |  |  |
| DiFilippo + Validation**^a^** | N/A | 0.7728 | 0.3133 | 0.0487 | 0.721 | 0.6593 |
| Piñero-Madrona**^b^** | 0.7728 | N/A | 0.2859 | 0.0431 | 0.5593 | 1 |
| PLUTTO**^c^** | 0.3133 | 0.2859 | N/A | 0.1656 | 0.5301 | 0.1977 |
| PLUTTO Validation**^d^** | 0.0487 | 0.0431 | 0.1656 | N/A | 0.0648 | 0.039 |
| PORTTLE**^e^** | 0.721 | 0.5593 | 0.5301 | 0.0648 | N/A | 0.5034 |
| SOLO-STUDY**^f^** | 0.6593 | 1 | 0.1977 | 0.039 | 0.5034 | N/A |
| N/A, not applicable. | | | | | | |

| **Table S2.** ROC curve analysis of selected total tumor load (TTL) thresholds for predicting ≥4 lymph node metastases in patients with HR+/HER2- breast cancer. | | | | | | | | | | | |
| --- | --- | --- | --- | --- | --- | --- | --- | --- | --- | --- | --- |
| **Threshold** | **Specificity** | | **Sensitivity** | **Accuracy** | **TN** | **TP** | **FN** | **FP** | **PPV** | **NPV** | **Youden** |
| 89,805.0 | 76.8 | | 58.2 | 73.9 | 2456 | 354 | 254 | 740 | 32.4 | 90.6 | 0.3507 |
| 90,600.0 | 76.9 | | 58.1 | 73.9 | 2458 | 353 | 255 | 738 | 32.4 | 90.6 | 0.3497 |
| 91,600.0 | 76.9 | | 58.1 | 73.9 | 2459 | 353 | 255 | 737 | 32.4 | 90.6 | 0.3500 |
| 93,000.0 | 77.0 | | 57.9 | 73.9 | 2460 | 352 | 256 | 736 | 32.4 | 90.6 | 0.3487 |
| 94,205.0 | 77.1 | | 57.7 | 74.0 | 2463 | 351 | 257 | 733 | 32.4 | 90.6 | 0.3480 |
| 94,705.0 | 77.1 | | 57.7 | 74.0 | 2465 | 351 | 257 | 731 | 32.4 | 90.6 | 0.3486 |
| 97,000.0 | 77.3 | | 56.9 | 74.0 | 2470 | 346 | 262 | 726 | 32.3 | 90.4 | 0.3419 |
| 99,500.0 | 77.3 | | 56.9 | 74.1 | 2472 | 346 | 262 | 724 | 32.3 | 90.4 | 0.3425 |
| 101,000.0 | 77.6 | | 56.4 | 74.2 | 2480 | 343 | 265 | 716 | 32.4 | 90.3 | 0.3401 |
| 102,400.0 | 77.7 | | 56.4 | 74.3 | 2483 | 343 | 265 | 713 | 32.5 | 90.4 | 0.3411 |
| 102,900.0 | 77.7 | | 56.4 | 74.3 | 2484 | 343 | 265 | 712 | 32.5 | 90.4 | 0.3414 |
| 103,100.0 | 77.8 | | 56.4 | 74.4 | 2487 | 343 | 265 | 709 | 32.6 | 90.4 | 0.3423 |
| 103,600.0 | 77.8 | | 56.4 | 74.4 | 2488 | 343 | 265 | 708 | 32.6 | 90.4 | 0.3426 |
| 104,500.0 | 77.9 | | 55.9 | 74.4 | 2490 | 340 | 268 | 706 | 32.5 | 90.3 | 0.3383 |
| 105,850.0 | 77.9 | | 55.8 | 74.4 | 2490 | 339 | 269 | 706 | 32.4 | 90.3 | 0.3367 |
| 106,850.0 | 77.9 | | 55.8 | 74.4 | 2491 | 339 | 269 | 705 | 32.5 | 90.3 | 0.3370 |
| 107,200.0 | 78.0 | | 55.8 | 74.4 | 2492 | 339 | 269 | 704 | 32.5 | 90.3 | 0.3373 |
| 107,550.0 | 78.0 | | 55.8 | 74.5 | 2494 | 339 | 269 | 702 | 32.6 | 90.3 | 0.3379 |
| 108,400.0 | 78.0 | | 55.6 | 74.4 | 2494 | 338 | 270 | 702 | 32.5 | 90.2 | 0.3363 |
| 109,550.0 | 78.0 | | 55.4 | 74.4 | 2494 | 337 | 271 | 702 | 32.4 | 90.2 | 0.3346 |
| 110,145.0 | 78.7 | | 55.3 | 74.9 | 2514 | 336 | 272 | 682 | 33.0 | 90.2 | 0.3392 |
| 110,455.0 | 78.7 | | 55.3 | 74.9 | 2515 | 336 | 272 | 681 | 33.0 | 90.2 | 0.3396 |
| 110,810.0 | 78.7 | 55.1 | | 74.9 | 2515 | 335 | 273 | 681 | 33.0 | 90.2 | 0.3379 |
| 111,350.0 | 78.8 | 55.1 | | 75.0 | 2517 | 335 | 273 | 679 | 33.0 | 90.2 | 0.3385 |
| 112,850.0 | 78.8 | 55.1 | | 75.0 | 2518 | 335 | 273 | 678 | 33.1 | 90.2 | 0.3388 |
| 114,500.0 | 78.8 | 55.1 | | 75.0 | 2519 | 335 | 273 | 677 | 33.1 | 90.2 | 0.3392 |
| 115,500.0 | 78.8 | 55.1 | | 75.1 | 2520 | 335 | 273 | 676 | 33.1 | 90.2 | 0.3395 |
| 201,000.0 | 82.7 | 46.9 | | 76.9 | 2642 | 285 | 323 | 554 | 34.0 | 89.1 | 0.2954 |
| 300,450.0 | 86.0 | 38.8 | | 78.5 | 2750 | 236 | 372 | 446 | 34.6 | 88.1 | 0.2486 |
| 401,500.0 | 87.5 | 34.0 | | 79.0 | 2797 | 207 | 401 | 399 | 34.2 | 87.5 | 0.2156 |
| 505,800.0 | 89.5 | 31.1 | | 80.2 | 2860 | 189 | 419 | 336 | 36.0 | 87.2 | 0.2057 |
| 600,415.0 | 90.6 | 28.0 | | 80.6 | 2895 | 170 | 438 | 301 | 36.1 | 86.9 | 0.1854 |
| 703,000.0 | 91.6 | 25.7 | | 81.1 | 2929 | 156 | 452 | 267 | 36.9 | 86.6 | 0.1730 |
| 800,550.0 | 92.3 | 22.9 | | 81.2 | 2949 | 139 | 469 | 247 | 36.0 | 86.3 | 0.1513 |
| 905,500.0 | 93.0 | 21.2 | | 81.5 | 2973 | 129 | 479 | 223 | 36.6 | 86.1 | 0.1424 |
| 1,000,215.0 | 93.6 | 19.9 | | 81.9 | 2993 | 121 | 487 | 203 | 37.3 | 86.0 | 0.1355 |
| 2,020,000.0 | 96.5 | 12.5 | | 83.1 | 3085 | 76 | 532 | 111 | 40.6 | 85.3 | 0.0903 |
| 3,100,205.0 | 97.8 | 8.1 | | 83.5 | 3127 | 49 | 559 | 69 | 41.5 | 84.8 | 0.0590 |
| 4,050,000.0 | 98.2 | 5.8 | | 83.4 | 3138 | 35 | 573 | 58 | 37.6 | 84.6 | 0.0394 |
| 5,115,355.0 | 98.8 | 4.6 | | 83.8 | 3159 | 28 | 580 | 37 | 43.1 | 84.5 | 0.0345 |
| 6,050,000.0 | 99.1 | 3.5 | | 83.8 | 3168 | 21 | 587 | 28 | 42.9 | 84.4 | 0.0258 |
| 7,053,950.0 | 99.4 | 2.5 | | 83.9 | 3176 | 15 | 593 | 20 | 42.9 | 84.3 | 0.0184 |
| 8,250,000.0 | 99.6 | 2.5 | | 84.0 | 3182 | 15 | 593 | 14 | 51.7 | 84.3 | 0.0203 |
| 9,135,000.0 | 99.6 | 2.1 | | 84.0 | 3184 | 13 | 595 | 12 | 52.0 | 84.3 | 0.0176 |
| 10,056,500.0 | 99.7 | 2.1 | | 84.1 | 3187 | 13 | 595 | 9 | 59.1 | 84.3 | 0.0186 |
| 12,058,500.0 | 99.7 | 1.3 | | 84.0 | 3188 | 8 | 600 | 8 | 50.0 | 84.2 | 0.0107 |
| 13,097,000.0 | 99.8 | 1.3 | | 84.0 | 3189 | 8 | 600 | 7 | 53.3 | 84.2 | 0.0110 |
| 14,050,000.0 | 99.8 | 0.8 | | 84.0 | 3189 | 5 | 603 | 7 | 41.7 | 84.1 | 0.0060 |
| 15,003,950.0 | 99.8 | 0.3 | | 83.9 | 3189 | 2 | 606 | 7 | 22.2 | 84.0 | 0.0011 |
| 16,800,000.0 | 99.8 | 0.2 | | 83.9 | 3190 | 1 | 607 | 6 | 14.3 | 84.0 | -0.0002 |
| 18,500,000.0 | 99.8 | 0.2 | | 83.9 | 3191 | 1 | 607 | 5 | 16.7 | 84.0 | 0.0001 |
| 19,066,090.0 | 99.9 | 0.2 | | 83.9 | 3192 | 1 | 607 | 4 | 20.0 | 84.0 | 0.0004 |
| 20,066,090.0 | 99.9 | 0.0 | | 83.9 | 3192 | 0 | 608 | 4 | 0.0 | 84.0 | -0.0013 |
| 24,280,000.0 | 99.9 | 0.0 | | 83.9 | 3193 | 0 | 608 | 3 | 0.0 | 84.0 | -0.0009 |
| 34,080,000.0 | 99.9 | 0.0 | | 84.0 | 3194 | 0 | 608 | 2 | 0.0 | 84.0 | -0.0006 |
| FN, false negatives; FP, false positives; NPV, negative predictive value; PPV, positive predictive value; ROC, receiver operating characteristic; TN, true negatives; TP, true positives. | | | | | | | | | | | |

| **Table S3.** ROC curve analysis of selected total tumor load (TTL) thresholds for predicting ≥4 lymph node metastases in patients with lobular HR+/HER2- breast cancer. | | | | | | | | | | |
| --- | --- | --- | --- | --- | --- | --- | --- | --- | --- | --- |
| **Threshold** | **Specificity** | **Sensitivity** | **Accuracy** | **TN** | **TP** | **FN** | **FP** | **PPV** | **NPV** | **Youden** |
| 88,155 | 76.1 | 64.8 | 73.8 | 267 | 57 | 31 | 84 | 40.4 | 89.6 | 0.4084 |
| 92,305 | 76.1 | 63.6 | 73.6 | 267 | 56 | 32 | 84 | 40.0 | 89.3 | 0.3970 |
| 98,500 | 76.1 | 62.5 | 73.3 | 267 | 55 | 33 | 84 | 39.6 | 89.0 | 0.3857 |
| 102,500 | 76.4 | 62.5 | 73.6 | 268 | 55 | 33 | 83 | 39.9 | 89.0 | 0.3885 |
| 103,100 | 76.6 | 62.5 | 73.8 | 269 | 55 | 33 | 82 | 40.1 | 89.1 | 0.3914 |
| 105,300 | 76.9 | 62.5 | 74.0 | 270 | 55 | 33 | 81 | 40.4 | 89.1 | 0.3942 |
| 108,700 | 77.5 | 62.5 | 74.5 | 272 | 55 | 33 | 79 | 41.0 | 89.2 | 0.3999 |
| 110,145 | 77.8 | 62.5 | 74.7 | 273 | 55 | 33 | 78 | 41.4 | 89.2 | 0.4028 |
| 115,145 | 78.1 | 62.5 | 74.9 | 274 | 55 | 33 | 77 | 41.7 | 89.3 | 0.4056 |
| 205,800 | 81.8 | 58.0 | 77.0 | 287 | 51 | 37 | 64 | 44.3 | 88.6 | 0.3972 |
| 301,355 | 83.8 | 47.7 | 76.5 | 294 | 42 | 46 | 57 | 42.4 | 86.5 | 0.3149 |
| 415,000 | 85.2 | 39.8 | 76.1 | 299 | 35 | 53 | 52 | 40.2 | 84.9 | 0.2496 |
| 509,000 | 86.0 | 39.8 | 76.8 | 302 | 35 | 53 | 49 | 41.7 | 85.1 | 0.2581 |
| 615,000 | 88.3 | 35.2 | 77.7 | 310 | 31 | 57 | 41 | 43.1 | 84.5 | 0.2355 |
| 715,000 | 89.2 | 31.8 | 77.7 | 313 | 28 | 60 | 38 | 42.4 | 83.9 | 0.2099 |
| 805,000 | 90.0 | 27.3 | 77.4 | 316 | 24 | 64 | 35 | 40.7 | 83.2 | 0.1730 |
| 910,600 | 90.9 | 26.1 | 77.9 | 319 | 23 | 65 | 32 | 41.8 | 83.1 | 0.1702 |
| 1,000,215 | 91.5 | 22.7 | 77.7 | 321 | 20 | 68 | 30 | 40.0 | 82.5 | 0.1418 |
| 2,004,200 | 96.0 | 10.2 | 78.8 | 337 | 9 | 79 | 14 | 39.1 | 81.0 | 0.0624 |
| 3,150,000 | 98.3 | 5.7 | 79.7 | 345 | 5 | 83 | 6 | 45.5 | 80.6 | 0.0397 |
| 4,130,000 | 98.9 | 4.5 | 80.0 | 347 | 4 | 84 | 4 | 50.0 | 80.5 | 0.0341 |
| 5,550,000 | 99.1 | 3.4 | 80.0 | 348 | 3 | 85 | 3 | 50.0 | 80.4 | 0.0255 |
| 6,100,000 | 99.1 | 2.3 | 79.7 | 348 | 2 | 86 | 3 | 40.0 | 80.2 | 0.0142 |
| 7,885,000 | 99.4 | 2.3 | 80.0 | 349 | 2 | 86 | 2 | 50.0 | 80.2 | 0.0170 |
| 11,585,000 | 99.7 | 2.3 | 80.2 | 350 | 2 | 86 | 1 | 66.7 | 80.3 | 0.0199 |
| 15,950,000 | 99.7 | 0.0 | 79.7 | 350 | 0 | 88 | 1 | 0.0 | 79.9 | -0.0028 |
| FN, false negatives; FP, false positives; NPV, negative predictive value; PPV, positive predictive value; ROC, receiver operating characteristic; TN, true negatives; TP, true positives. | | | | | | | | | | |

| **Table S4.** Univariate and multivariate logistic regression models for predicting the presence of four or more metastatic lymph nodes in patients with HR+/HER2- breast cancer, n=3041. | | | | | | | | | |
| --- | --- | --- | --- | --- | --- | --- | --- | --- | --- |
|  |  | **Univariate Model** | | | | **Multivariate Model** | | | |
| **Variable** | **Levels** | **OR** | **Lower 95% CI** | **Upper 95% CI** | ***P*-value** | **OR** | **Lower 95% CI** | **Upper 95% CI** | ***P*-value** |
| Estrogen receptors | Positive vs Negative | 1.27 | 0.44 | 5.42 | 0.6955 |  |  |  |  |
| Progesterone receptors | Positive vs Negative | 0.93 | 0.71 | 1.23 | 0.5897 |  |  |  |  |
| Age (years) |  | 1 | 0.99 | 1 | 0.2364 |  |  |  |  |
| Ki67 (%) | >= 20 vs < 20 | 1.2 | 0.94 | 1.53 | 0.1381 |  |  |  |  |
| Number of non-sentinel nodes removed | | 1.05 | 1.04 | 1.06 | <0.0001 | 1.03 | 1.01 | 1.04 | 0.0004 |
| Tumor grade (num) |  | 1.64 | 1.42 | 1.89 | <0.0001 | 1.38 | 1.16 | 1.64 | 0.0003 |
| Maximum tumor diameter (mm) | | 1.03 | 1.02 | 1.03 | <0.0001 | 1.02 | 1.01 | 1.02 | 0.0001 |
| Lymphovascular invasion | Yes vs No | 2.75 | 2.29 | 3.31 | <0.0001 | 1.84 | 1.48 | 2.27 | <0.0001 |
| Total Tumor Load (log_10_) |  | 1.36 | 1.31 | 1.41 | <0.0001 | 1.32 | 1.27 | 1.38 | <0.0001 |
| CI, confidence interval. |  |  |  |  |  |  |  |  |  |

| **Table S5.** ROC curve analysis of the multivariate model for predicting four or more metastatic lymph nodes in patients with HR+/HER2- breast cancer. | | | | | | | | | | |
| --- | --- | --- | --- | --- | --- | --- | --- | --- | --- | --- |
| **Threshold (log_10_)** | **Specificity** | **Sensitivity** | **Accuracy** | **TN** | **TP** | **FN** | **FP** | **PPV** | **NPV** | **Youden** |
| 0.150828620 | 65.1 | 77.7 | 67.1 | 1656 | 386 | 111 | 888 | 30.3 | 93.7 | 0.4276 |
| 0.150868814 | 65.1 | 77.7 | 67.2 | 1657 | 386 | 111 | 887 | 30.3 | 93.7 | 0.4280 |
| 0.151103480 | 65.2 | 77.7 | 67.2 | 1658 | 386 | 111 | 886 | 30.3 | 93.7 | 0.4284 |
| 0.151386706 | 65.2 | 77.5 | 67.2 | 1658 | 385 | 112 | 886 | 30.3 | 93.7 | 0.4264 |
| 0.151475172 | 65.2 | 77.5 | 67.2 | 1659 | 385 | 112 | 885 | 30.3 | 93.7 | 0.4268 |
| 0.151576454 | 65.3 | 77.5 | 67.2 | 1660 | 385 | 112 | 884 | 30.3 | 93.7 | 0.4272 |
| 0.151670697 | 65.3 | 77.5 | 67.3 | 1661 | 385 | 112 | 883 | 30.4 | 93.7 | 0.4276 |
| 0.151806299 | 65.3 | 77.5 | 67.3 | 1662 | 385 | 112 | 882 | 30.4 | 93.7 | 0.4279 |
| 0.152123343 | 65.4 | 77.5 | 67.4 | 1664 | 385 | 112 | 880 | 30.4 | 93.7 | 0.4287 |
| 0.152395351 | 65.4 | 77.5 | 67.4 | 1665 | 385 | 112 | 879 | 30.5 | 93.7 | 0.4291 |
| 0.152524433 | 65.5 | 77.5 | 67.4 | 1666 | 385 | 112 | 878 | 30.5 | 93.7 | 0.4295 |
| 0.152715996 | 65.5 | 77.3 | 67.4 | 1666 | 384 | 113 | 878 | 30.4 | 93.6 | 0.4275 |
| 0.152861369 | 65.5 | 77.3 | 67.4 | 1667 | 384 | 113 | 877 | 30.5 | 93.7 | 0.4279 |
| 0.152888251 | 65.6 | 77.3 | 67.5 | 1668 | 384 | 113 | 876 | 30.5 | 93.7 | 0.4283 |
| 0.152922195 | 65.6 | 77.1 | 67.4 | 1668 | 383 | 114 | 876 | 30.4 | 93.6 | 0.4263 |
| 0.153288978 | 65.6 | 77.1 | 67.5 | 1670 | 383 | 114 | 874 | 30.5 | 93.6 | 0.4271 |
| 0.153722419 | 65.7 | 77.1 | 67.5 | 1671 | 383 | 114 | 873 | 30.5 | 93.6 | 0.4275 |
| 0.153881379 | 65.7 | 77.1 | 67.6 | 1672 | 383 | 114 | 872 | 30.5 | 93.6 | 0.4279 |
| 0.154045430 | 65.7 | 76.9 | 67.5 | 1672 | 382 | 115 | 872 | 30.5 | 93.6 | 0.4258 |
| 0.154229049 | 65.7 | 76.7 | 67.5 | 1672 | 381 | 116 | 872 | 30.4 | 93.5 | 0.4238 |
| 0.154442894 | 65.8 | 76.7 | 67.5 | 1673 | 381 | 116 | 871 | 30.4 | 93.5 | 0.4242 |
| FN, false negatives; FP, false positives; NPV, negative predictive value; PPV, positive predictive value; ROC, receiver operating characteristic; TN, true negatives; TP, true positives. | | | | | | | | | | |
